# Cytotoxicity and Preliminary Analysis of the Pro-apoptotic and Cell Cycle Arrest Effects of *Lantana ukambensis* Against Colorectal Cancer Cells

**Published:** 2020-08-07

**Authors:** Wamtinga Richard Sawadogo, Yun Luo, Bethany Elkington, Tong-Chuan He, Chong-Zhi Wang, Chun-Su Yuan

**Affiliations:** 1 Tang Center for Herbal Medicine Research, Pritzker School of Medicine, University of Chicago, Chicago, Illinois, 60637, USA; 2 Department of Anesthesia and Critical Care, Pritzker School of Medicine, University of Chicago, Chicago, Illinois, 60637, USA; 3 Institut de Recherche en Sciences de la Santé (IRSS/CNRST), 03 BP 7192, Ouagadougou 03, Burkina Faso; 4 Key Laboratory of Modern Preparation of Traditional Chinese Medicine, Ministry of Education, Jiangxi University of Traditional Chinese Medicine, Nanchang, 330004, China; 5 College of Pharmacy, University of Illinois at Chicago, Chicago, Illinois, 60612, USA; 6 Department of Orthopedic Surgery and Rehabilitation Medicine, Pritzker School of Medicine, University of Chicago, Chicago, Illinois, 60637, USA

**Keywords:** Medicinal plants, Cancer, Cytotoxicity, Apoptosis, Cell cycle arrest

## Abstract

*Lantana ukambensis* (Vatke) Verdc. (Verbenaceae) is a seasonal herb widely spread in the West African region. The whole plant is used for the treatment of wounds, infections, and inflammatory pathologies. The purpose of this research is to evaluate the cytotoxicity and to analyze the probable pro-apototic, and cell cycle arrest effects of *L. ukambensis* methylene chloride extract and its fractions against HCT-116 and HT-29 colorectal cancer cells using preliminary tests in order to highlight the interest of this plant in the search of new anticancer molecules.

The dried powder of the whole plant was extracted by methylene chloride maceration for 24 hours and the extract was divided into five fractions. The cytotoxicity of the crude extract and fractions were evaluated by the MTS assay. The most active fractions were subjected to some preliminary assays including crystal violet, Hoechst staining, cell cycle arrest, and annexin V/PI assays on the cancer cells to highlight the probable mechanism of action of these fractions.

The methylene chloride, ethyl acetate, and 1-butanol fractions of *L. ukambensis* crude extract demonstrated significant antiproliferative effects on HCT-116 and HT-29 cell growth with IC_50_ values ranging between 2 to 15 μg/mL. 1-butanol and ethyl acetate fractions decreased the G1 phase by 20.53% and 28.47% and increased the G2/M by 23.47% and 25.90% respectively on HCT-116. Moreover, 1-butanol fraction increased the cumulative value of apoptotic cells by 49.77% on HCT-116 and ethyl acetate fraction increased this value by 53.37% at 15 μg/mL after 48 hours of exposure.

The outcome of this study suggests the potential of 1-butanol and ethyl acetate fractions for the isolation of anticancer molecules against colorectal cancer.

## Introduction

*Lantana ukambensis* (Vatke) Verdc. (Verbenaceae) is a seasonal herb widely spread in the West African region, especially in areas with a tropical climate [1]. Its red fruits are widely eaten by children in the countryside whilst the whole plant is used in traditional medicine for the management of many diseases. In Burkina Faso’s folk medicine, *L. ukambensis* whole plant is used for the treatment of wounds, infections, and inflammatory pathologies [1]. Our previous studies demonstrated the antioxidant, antiproliferative, and antileishmanial activities of *L. ukambensis* as a scientific base of its traditional uses [1,2,3,5]. The World Health Organization (WHO) estimates that more than 80% of the African population use traditional medicine for their primary health care. These traditional uses are justified by cultural and economic reasons [4,5]. Plants are the most important component of this traditional medicine and constitute an important source of therapeutic compounds to improve the population’s quality of health [4,6,7,8].

Among the most dreaded pathologies in the world, cancer is one of the most difficult to overcome due to the multiple types, forms, and various causes, as well as frequent chemo-resistance. Cancer is considered to be the second leading cause of death in the world with an estimated number of 18.1 million new cases and 9.6 million deaths in 2018 [9]. Unfortunately, the number of new cancer cases per year is expected to rise to 23.6 million by 2030. Among the most common cancers, the WHO estimates that colon cancer occupies the third position after lung and breast cancers, with 1.80 million cases reported in 2018 [9,10].

Conventional medicine, as well as traditional medicine, offer treatments for cancer patients. However these therapies are not always safe. Indeed, conventional chemotherapeutic drugs, especially from synthetic origin, target the receptor of both normal and cancer cells due to non-selective toxicity and may induce severe side effects and high toxicity leading to a deterioration in the quality of life of patients [11]. On the other side, traditional healers often have a different view about the physiopathology of cancer, the doses of administration, and the level of toxicity of their plant extracts, which can lead to long-term toxicity including liver toxicity, genotoxicity, kidney toxicity, and gastro-intestinal disturbances [12,13].

For all of these reasons, the research of new anticancer drugs is more and more focused on natural compounds, especially from medicinal plants. In fact, it’s estimated that at least 60% of new anticancer molecules have been derived from natural compounds [8]. Our previous studies revealed that *L. ukambensis* is a potential source of new anticancer molecules. Indeed, we demonstrated the cytotoxic effect of the methylene chloride extract of this plant against cancer cell lines including KB, K562, U937, Jurkat and Raji with less toxicity on healthy peripheral blood mononuclear cells (PBMCs) [1]. In addition, the phytochemical screening showed the presence of saponins, flavonoids, tannins, triterpenoids, and steroids in the extract of *L. ukambensis.* These compounds are well-known for their anticancer properties. Recently, we isolated one compound, 5-hydroxy-6,7,3’,4’,5’-pentamethoxyflavone, which showed significant antiproliferative and pro-apoptotic effect against U937 cells [1].

The purpose of this research is to evaluate the cytotoxicity and to analyze the probable pro-apototic, and cell cycle arrest effects of *L. ukambensis* methylene chloride extract and its fractions against HCT-116 and HT-29 colorectal cancer cells using preliminary tests in order to highlight the interest of this plant in the search of new anticancer molecules.

## Materials and Methods

### Chemicals

The solvents used in this study were obtained from Fisher Scientific (Norcross, GA) with HPLC grade; cell culture medium (McCoy’s 5A, Leibovitz’s L-15) and serum (FBS) from Mediatech, Inc. (Herndon, VA); antibiotics (penicillin and streptomycin) from Sigma-Aldrich (St. Louis, MO, USA); Milli-Q deionized water (18 MΩ-cm) system from Millipore (Milford, MA, USA); The reagents (MTS assay kit, CellTiter for Cell Proliferation Assay Promega (Madison, WI, USA); FITC-Annexin V kit from BD Biosciences (Rockville, MD, USA); PI/RNase staining buffer from BD Biosciences Pharmingen (San Diego, CA, USA); cell culture plastic materials from Falcon Labware (Franklin Lakes, NJ, USA).

### Plant Material

The whole plant of *L. ukambensis* (Vatke) Verdc. (Verbenaceae) was collected by Sawadogo WR in August, 2018 by Bobo Dioulasso (Burkina Faso) with the following GPS position: Latitude (dd) of 17.22320 and Longitude (dd) of −7.25314. The plant was previously collected in the same area and identified in 2010 by Professor Millogo Jeanne at the Laboratory of Ecology, University Joseph Ki-Zerbo of Ouagadougou, Burkina Faso. The identification was confirmed by Professor Amadé Ouedraogo, botanist at the Laboratory of Ecology, University Joseph Ki-Zerbo of Ouagadougou. A voucher specimen was deposited at the herbarium of the National center for scientific research and technology of Burkina Faso under the specimen number LU-003.

### Extraction and Fractionation

An amount of 100g of *L. ukambensis* powder was submitted to maceration in methylene chloride (500mL) with stirring during 24 hours. After filtration, the extract was evaporated and lyophilized to obtain dried extract (7.5g). An amount of 2g of the crude extract was dissolved in 10 mL of DMSO plus 90 mL of distilled water for the liquid-liquid fractionation with the following solvents: Petroleum ether (PE), methylene chloride (MC), ethyl acetate (EA), and 1-buthanol (1B). The five fractions were dried through evaporation and lyophilization and the dried extracts was used for biological assays.

### Cell Culture

Colorectal cancer cells (HCT-116, HT-29) from the American Type Tissue Collection (Rockville, MD, USA) were cultured in McCoy's 5A, added by 10% FBS, penicillin (100IU/ml), and streptomycin (100μg/ml). HCT-116 and HT-29 were incubated at 37°C in 5% CO_2_ environment and sub-cultured every two days according to the protocol described by Wang et al, 2006 [14]. Briefly, when the cells covered 90% of the flask’s surface area with free of contamination, they are collected by adding 1mL of trypsin and 2mL of collected cells are re-suspended into a new flask containing 8mL of culture medium.

### Cell Proliferation Analysis

The MTS assay was used to analyze the effect of *L. ukambensis* on colorectal cancer cells proliferation as described by Wang et al [14]. The plant extract and fractions were dissolved in DMSO and stored at 4°C before use. HCT-116 and HT-29 cells were placed in a 96-well plate and incubated at 37°C plus 5% CO_2_. After 24 horns on incubation, the medium in each well was replaced by 200 μL of new culture medium and the tested samples at different concentrations were added to the wells. The crude extract was tested at concentrations ranged from 10 to 300μg/mL after 48 hours in order to verify the level of its cytotoxicity against colorectal cancer cells. The fractions of this crude extract were tested at 5 and 50μg/mL after 48 hours of exposure to select the most cytotoxic fractions. After this selection, the antiproliferative effect was performed at 1, 2, 4, 5, 8, 10μg/mL after 24, 48 and 72 hours of exposure in order to determine the IC_50_ values of these fractions. Control wells received culture medium plus the same quantity of DMSO without drugs. In order to avoid false results, the final concentration of DMSO in tested groups did not exceed 1%. Wells containing medium but no cells are considered as blank. After 48 hours of exposure, the medium in each well was replaced by 100μL of fresh medium plus 20μL of CellTiter 96 solution. This reagent in contact with viable cells is bio-reduced by dehydrogenase enzymes into a formazan product. The number of living cells is determined by measuring the quantity of formazan product which correspond to the amount of absorbance at 490nm. After 2 hours of incubation; 60μL of medium from each well was transferred to an ELISA 96-well plate, and the absorbance of the formazan product at 490 nm was measured. Results were expressed as percent of control (DMSO vehicle set at 100%) from three independents assays.

### Crystal Violet Staining Assay

The crystal violet staining assay was performed as described by Wan et al, 2018 [15]. Briefly, the cancer cells were placed in 24-well plates at 100 cells/well.

After 24 h of incubation, the medium was removed and replaced by fresh culture medium plus different concentrations of the most cytotoxic fractions of *L. ukambensis*. The tested concentrations (5, 10 and 15μg/mL) were chosen according to the IC_50_ values of the cytotoxic fractions. After 48 hours of exposure, the cells were washed and stained with 0.2% crystal violet in 10% phosphate-buffered formaldehyde for 2 min. Cells were then washed with PBS and the adherent cells at the bottom of each well were photographed under a microscope in order to analyze morphological changes in the cytoplasmic vacuoles of each cell.

### Preliminary Cell Cycle Analysis

The effect of the active fractions of *L. ukambensis* on the cell cycle phases was analyzed, especially on the G1, S, and G2/M phases, as described by Yao et al, 2018 [16]. HCT-116 and HT-29 cells were incubated in 24-well plates for 24 hours. The cell culture medium was removed and replaced by fresh medium plus different concentrations of plant fractions and incubated for 48 hours. These concentrations (5, 10 and 15μg/mL) were chosen according to the IC_50_ values of the fractions. The cells were then collected by centrifugation (2000 rpm / 5 minutes) and fixed by 100% ethanol at 4°C for 2 hours. After treatment with 0.25% Triton X-100 for 5 min on an ice bath, each cell line was re-suspended in 300μ1 of PBS, 40μg/ml propidimn iodide (PI) and 0.1mg/ml RNase and incubated in the dark at room temperature for 20 min. Cell cycle analysis was performed using a LSRII flow cytometer 585 mn (BD Biosciences, Franklin Lakes, NJ) and FlowJo 10.1.0 software (Tree Star, Ashland, OR, USA). In order to increase the level of accuracy, at least 20,000 cells were counted for each measurement.

### Preliminary Pro-apoptosis Analysis

Two methods were performed for the preliminary analysis of the pro-apoptotic effect of *L. ukambensis* extract and fractions: Hoechst 33258 staining and Annexin V/ Propidium Iodide assays as described in our previous works [1,14].

#### Hoechst 33258 Staining Assay

The Hoechst 33258 staining assay was performed as previously described by Sawadogo et al., 2015 [1] in order to analyze, by fluorescence microscope at 365 nm, the nuclear fragmentation in cells treated by cytotoxic agent. Briefly, the colorectal cancer cells were seeded in a 24-well plate at 100,000 cells/well and treated with different concentrations of the plant extracts for 48 hours. These concentrations (5, 10 and 15μg/mL) were chosen according to the IC_50_ values of the fractions. In each well, 1mL of trypsin was added to remove the cells for centrifugation at 2000 rpm / 5 min. Then the cells were collected and stained with O.Olmg/mL Hoechst 33258, 33mg/mL fonnalhehyde, and 5mg/mL NP-40 in PBS for 10 min in the dark. Finally, the nuclear fragmentation in treated cells was observed and photographed using fluorescence microscopy.

#### Annexin V/ Propidium Iodide staining (PI) assays

Annexin V is a Ca^2+^-dependent phospholipid-binding protein that binds strongly to phosphatidylserine residues translocated to the outside of the apoptotic cells at the early stage of apoptosis. Then, cell apoptosis can be assayed by flow cytometry after staining with annexin V. Briefly, cells were seeded in 24-well plates at the concentration of 200,000 cells/well for 48 hours. The medium was removed and replaced by fresh one and the plant extracts were measurement. added in the treated wells at 5, 10 and 15μg/mL. These concentrations were chosen according to the IC_50_ values of the fractions. After 48 hours of exposure, cells floating in the medium were harvested using trypsin and centrifugation of 5 min at 600g. The collected cells were stained with annexin-V FITC and propidium iodide (PI), according to the manufacturer’s instructions. Cells in medium culture without extracts were used as a control for double staining. Stained samples were analyzed by FACScan flow cytometer (Becton Dickinson, Mountain View, CA) and Flow Jo software (Tree Star, Ashland, OR). In order to increase the level of accuracy, at least 20,000 cells were counted for each measurement.

### Statistical Analysis

Data are presented as the mean of at least three independent assays (M±SD). A two-way ANOVA of multiple comparisons from GraphPad Prism 6 was employed to determine whether the results had statistical significance. In some cases, the student’s t-test was used for comparing two groups. The difference is significance at p<0.05. In each figure, one star (*) corresponds to the p<0.05, 2 stars (**) for p<0.01, 3 stars (***) for p<0.001, and 4 stars (****) for p<0.0001

## Results

### Cytotoxic Effect

**Figure 1:**
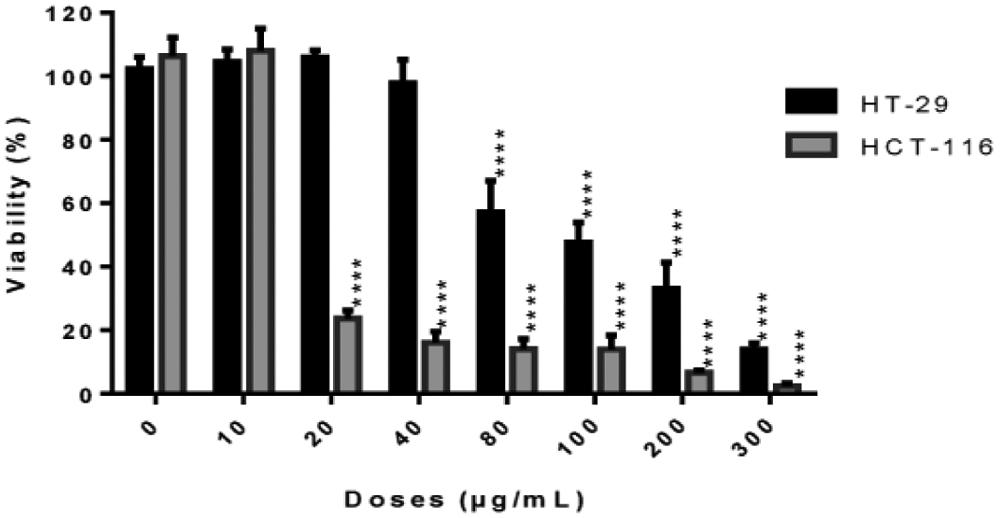
Cytotoxic effect of the crude methylene chloride extract of *L. ukambensis* on HCT-116 and HT-29 at concentrations ranged from 10 to 300μg/mL after 48 hours of exposure. The results are the mean ± SD of three independent assays. The level of statistical significance was set at p<0.05. In each figure, one star (*) corresponds to the p<0.05, 2 stars (**) for p<0.01, 3 stars (***) for p<0.001, and 4 stars (****) for p<0.0001.

The crude methylene chloride extract of *L. ukambensis* showed significant cytotoxic effect against both colorectal cancer cell lines after 48 hours of treatment at 20μg/mL for HCT-116 and 80μg/mL for HT-29. (Figure 1).

**Figure 2:**
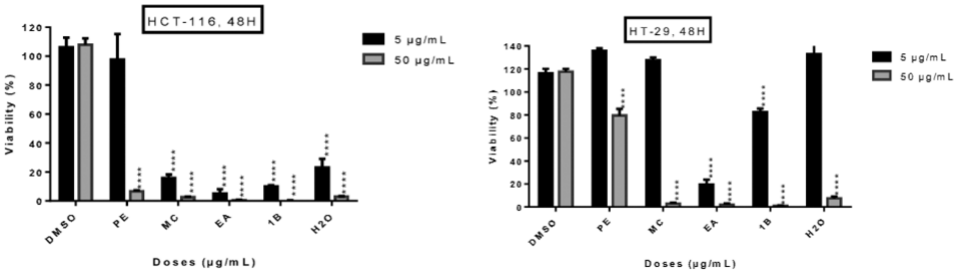
Cytotoxic effect of five fractions from crude methylene chloride extract of *L. ukambensis* on HCT-116 and HT-29 at 5 and 50μg/mL after 48 hours of exposure. PE=Petroleum ether, MC= Methylene choride, EA= Ethyl acetate, 1B= 1-Butanol, H2O= Water, DMSO=Dimethylsulfoxyde. The results are the mean ± SD of three independent assays. The level of statistical significance was set at p<0.05. In each figure, one star (*) corresponds to the p<0.05, 2 stars (**) for p<0.01, 3 stars (***) for p<0.001, and 4 stars (****) for p<0.0001.

**Figure 3:**
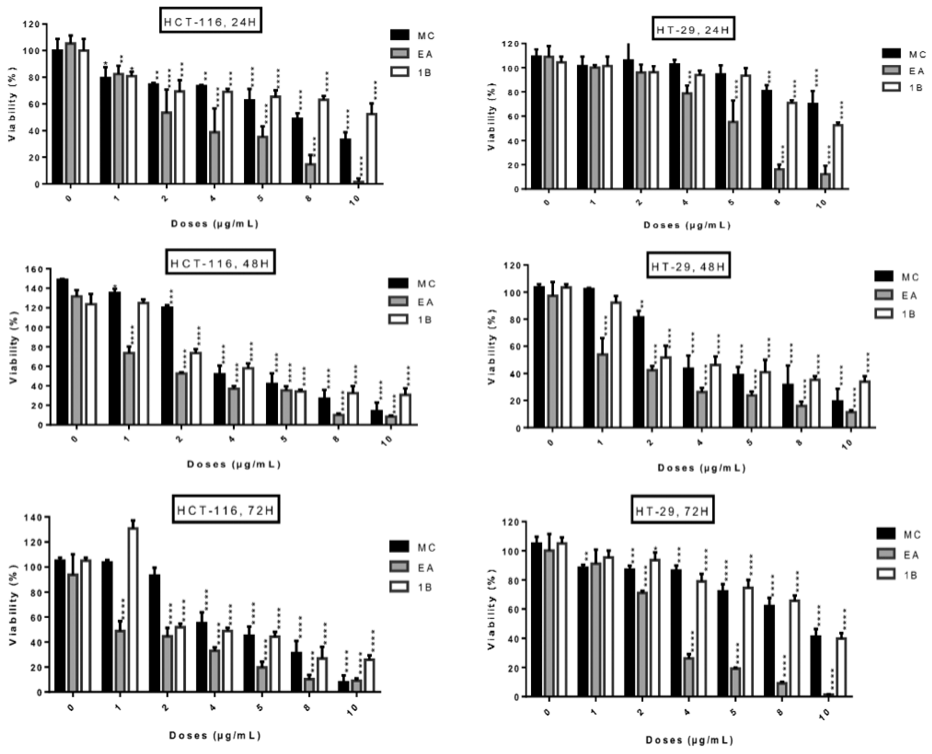
Cytotoxic effect of active fractions (MC, EA and 1B) from *L. ukambensis* on HCT-116 and HT-29 at 1, 2, 4, 5, 8 and 10μg/mL after 24, 48, and 72 hours of exposure. The results are the mean ± SD of three independent assays. The level of statistical significance was set at p<0.05. In each figure, one star (*) corresponds to the p<0.05, 2 stars (**) for p<0.01, 3 stars (***) for p<0.001, and 4 stars (****) for p<0.0001.

The five fractions from *L. ukambensis* crude extract demonstrated significant cytotoxicity against both cell lines at 50μg/mL after 48 hours of exposure (Figure 2). At 5μg/mL, the most cytotoxic fractions are those from methylene chloride, ethyl acetate, and 1-butanol (Figure 2). These most cytotoxic fractions were tested at different concentrations and time points in order to determine their IC_50_ values. All of these fractions showed significant cytotoxic effect on HCT-116 and HT-29 cells (Figure 3) in a dose dependent manner.

The concentrations of *L. ukambensis* crude extract and its most cytotoxic fractions, which inhibited 50% of cancer cells proliferation (IC_50_), are indicated in table 1. Among them, the ethyl acetate fraction is found to be the most cytotoxic. Indeed, this fraction demonstrated ten times more cytotoxicity on HCT-116 and twenty times on the HT-29 cells, when compared to the crude extract.

### Crystal violet staining

**Figure 4:**
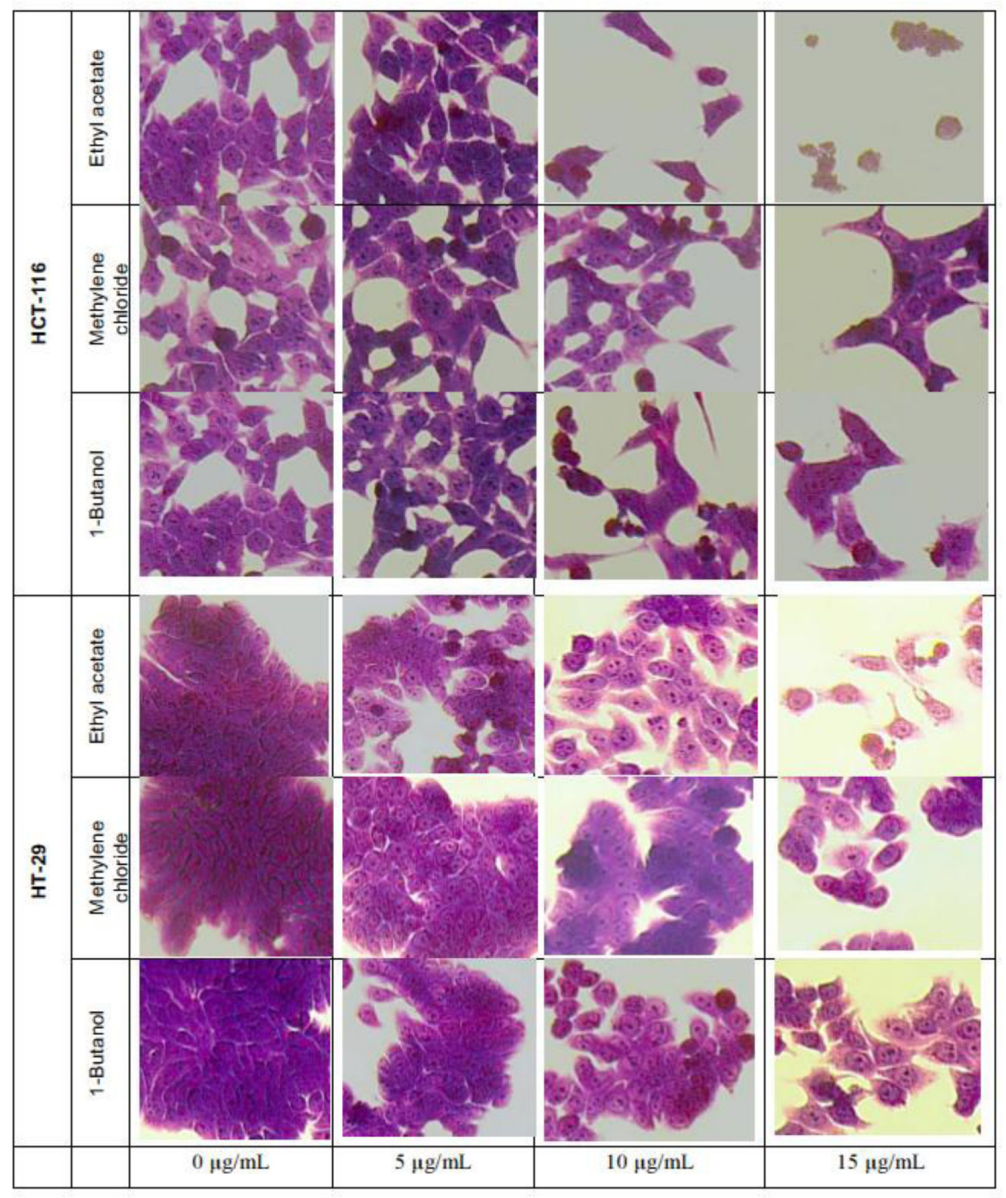
HCT-116 and HT-29 cells treated with the cytotoxic fractions of *L. ukambensis* at 5, 10, and 15μg/mL for 48 hours. The cells were stained by crystal violet, and pictures were made using Olympus IX70 microscope (X20). Morphological damages are signs of cytotoxicity of the fractions.

The crystal violet method allows cell morphology observation by microscope, when treated with *L. ukambensis* fractions. The results showed that at 48 hours of exposure, the three fractions (methylene chloride, 1 -butanol, and ethyl acetate) induced damages on both cancer cells morphology (HCT-116 and HT-29) until cell death (Figures 4) at 15μg/mL. Results were confirmed by three independent analyses. The effect of these fractions is in a dose dependent manner.

### Preliminary cell cycle analysis

**Figure 5:**
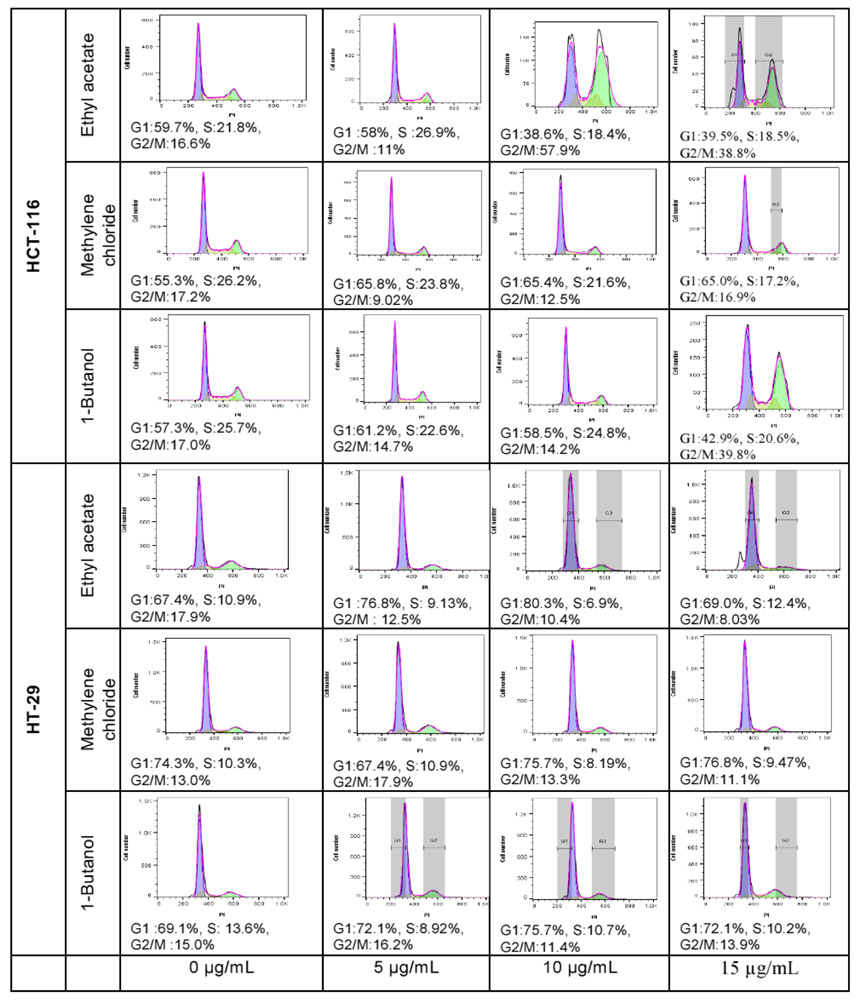
Cell cycle analysis by cytometry of HCT-116 and HT-29 cells treated with *L. ukambensis* fractions at 5, 10, and 15μg/mL for 48 hours. G1 phase is represented by the first pic in blue color, S phase is the middle one in yellow and G2/M the last pic in green color.

**Figure 6:**
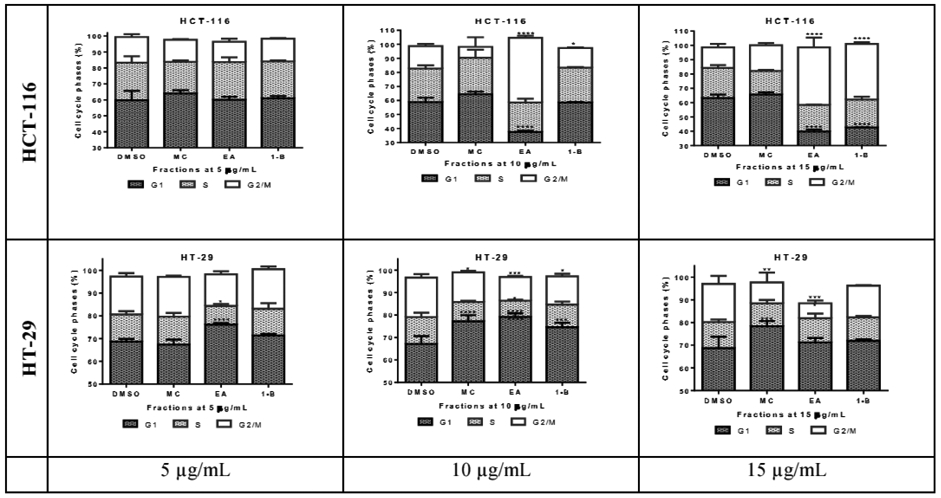
Quantitative analysis of cell cycle phases S, G1, and G2/M in HCT-116 and HT-29 cells treated with Ethyl acetate. Methylene chloride, and 1-Butanol fractions from *Lantana ukambensis* at 5, 10, 15μg/mL for 48 hours. The results are the mean ± SD of three independent analysis. The level of statistical significance was set at p<0.05. In each figure, one star (*) corresponds to the p<0.05, 2 stars (**) for p<0.01, 3 stars (***) for p<0.001, and 4 stars (****) for p<0.0001.

Cell cycle analysis is performed to assess the impact of the cytotoxic fractions on the different phases of the cell cycle, including G1, S, and G2/M. The results were confirmed by three independent analyses (Figure 5). At 15μg/mL, 1-butanol fraction induced a decrease in G1 phase of 20.53% and an increase of 23.47% in G2/M phases on HCT-116 after 48 hours of exposure, whilst the inverse effects were observed against HT-29; where the G1 phase was increased by 24.6% and G2/M decreased by 2.90%. In the same condition, the ethyl acetate fraction decreased the G1 phase by 28.47% and increased the G2/M by 25.90% on HCT-116 whilst on HT-29 the G1 phase was lightly increased by 2.5% and the G2/M decreased by 10.26% (Figure 6). In both cell lines, the fractions have no significant effect on the S phase. The methylene chloride fraction did not show significant effect on the cycle cell of either colorectal cancer cells.

### Preliminary Pro-apoptosis Analysis

**Figure 7:**
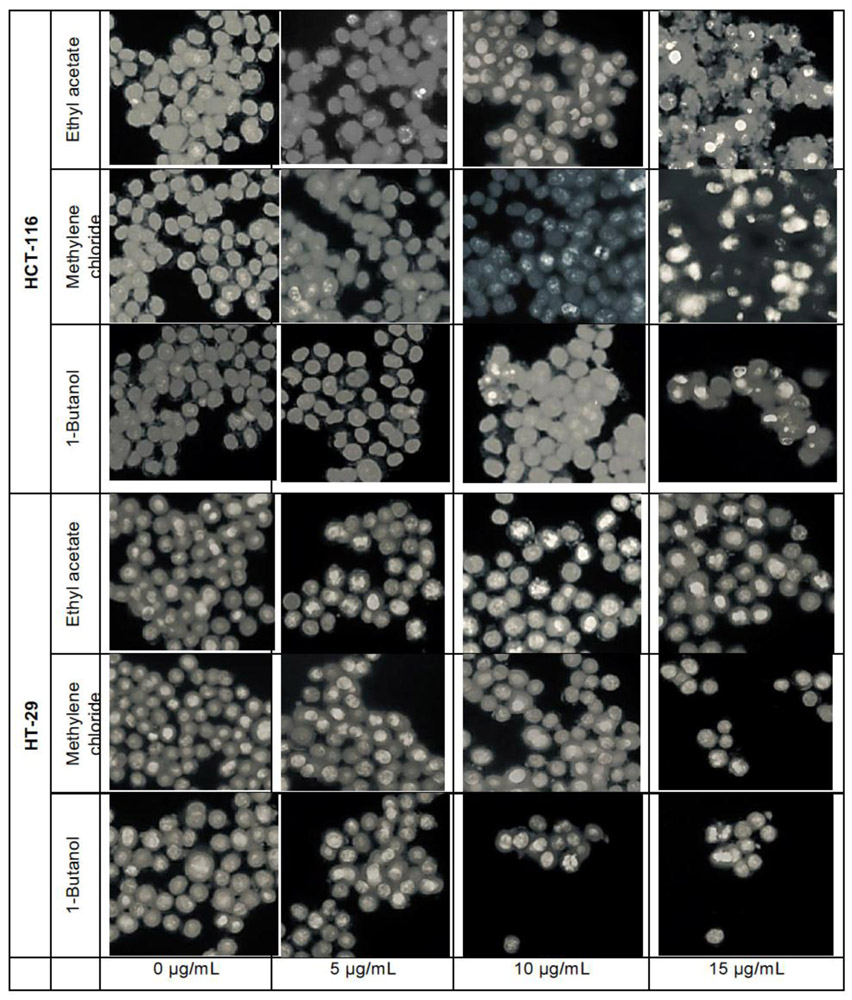
HCT-116 and HT-29 cells treated with the cytotoxic fractions of *L. ukambensis* at 5, 10, and 15μg/mL for 48 hours. The cells were stained by Hoechst, and pictures were made using an Olympus IX70 microscope (X20). Nucleus fragmentation is the sign of apoptosis.

**Figure 8:**
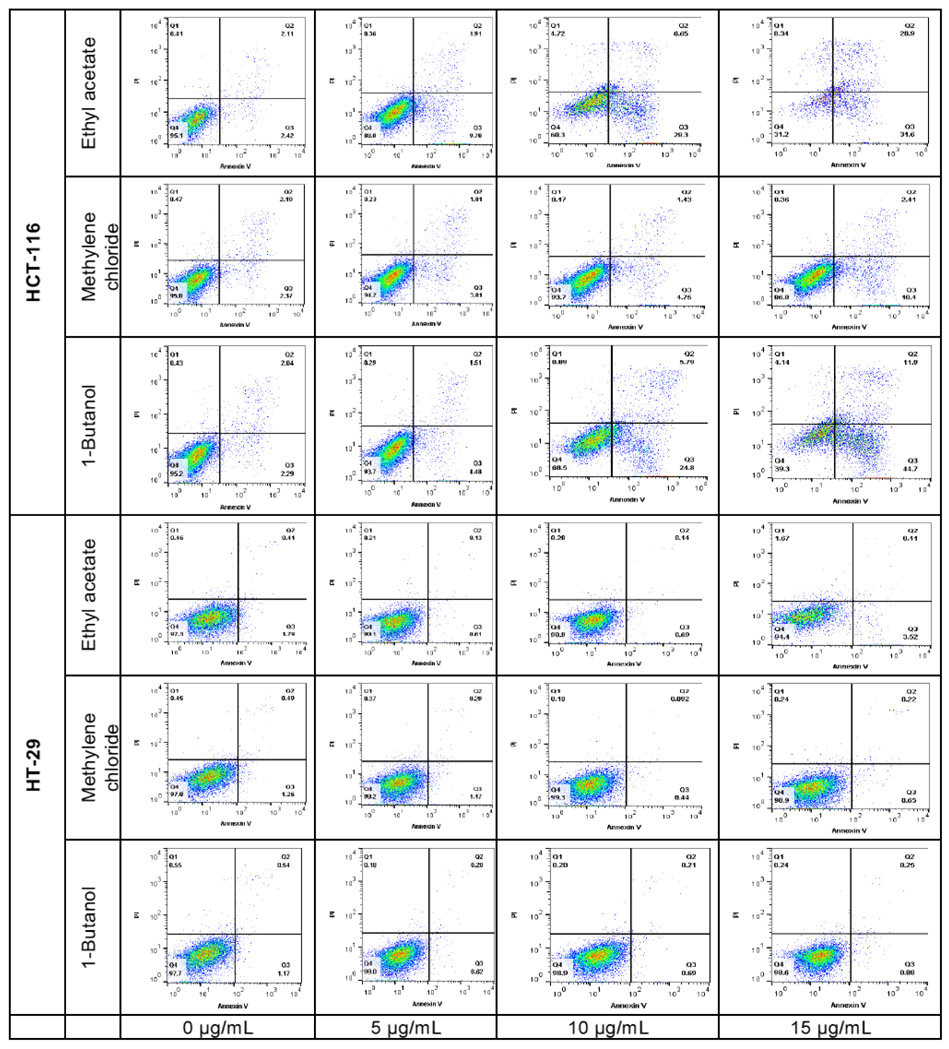
Apoptosis analysis by cytometry of HCT-116 and HT-29 cells treated with *L. ukambensis* fractions at 48 hours. The apoptotic cells are observed in the right side of the square, including early apoptosis at the bottom, and late apoptosis in the upper, whilst the living cells are at the bottom left side of the square and necrosis in the upper.

**Figure 9:**
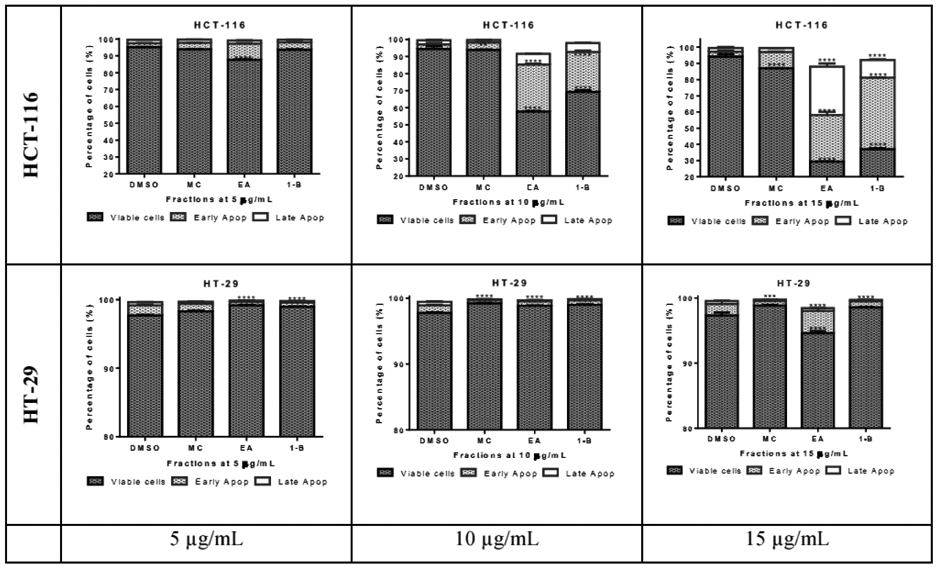
Quantitative analysis of early and late apoptosis in HCT-116 and HT-29 treated with Ethyl acetate, Methylene chloride, and 1-Butanol fractions from *Lantana ukambensis* at 5, 10, and 15μg/mL for 48 hours. The results are the mean ± SD of three independent analysis. The level of statistical significance was set at p<0.05. In each figure, one star (*) corresponds to the p<0.05, 2 stars (**) for p<0.01, 3 stars (***) for p<0.001, and 4 stars (****) for p<0.0001.

The pro-apoptotic effect of the most active fractions was evaluated by Hoechst 33258 staining and Annexin V/PI assays. Nucleus fragmentation is the sign of apoptosis when using the Hoechst assay (Figure 7). In the Annexin V/PI assay, the apoptotic cells are observed in the right side of the square, including early apoptosis at the bottom, and late apoptosis in the upper, whilst the living cells are at the bottom left side of the square and necrosis in the upper (Figure 8). Results of both assays were confirmed by three independent analyses. Significant apoptotic HCT-116 cells were observed at 48 hours in wells treated by 1-butanol and ethyl acetate fractions, whilst at the same conditions, no significant apoptotic effect was induced on HT-29 (Figures 7 and 8). In the Annexin V/PI assay, the 1-butanol and ethyl acetate fractions also showed significant apoptotic effect on HCT-116 at 15 μg/mL (Figures 8). Indeed, the 1-butanol fraction induced an increase of the cumulative value of early and late apoptosis by 49.77% on HCT-116 and the ethyl acetate increased this value by 53.37% at 15 pg/mL after 48 hours of exposure (Figure 9).

## Discussion

The results of this study show that the 1-butanol and ethyl acetate fractions of *L. ukambensis* are highly cytotoxic to colorectal cancer cells. In general, the HCT-116 cell line is more sensitive to cytotoxic agents compared to HT-29 justifying the fact that the IC_5_o values of the tested fractions are lowest on HCT-116. Interestingly, our previous research demonstrated that the extracts and fractions from *Lantana ukambensis* induce low toxicity against healthy cells (Vero, MCR-5 and PBMCs) compared to cancer cell lines (KB, K562, U937, Jurkat and Raji) [1,3]. The ethyl acetate is a polar aprotic solvent whilst the 1-butanol has a role as a protic solvent [17,18]. These type of solvents may contain polar molecules like saponins and polyphenols including tannins, anthocyanins, phenolic acids, flavonoids, and stilbenes [19,20,21]. All these compounds are well known to possess preventive and curative benefits against cancers [22,23,24,25]. Very little research has been published on the anticancer properties of *L. ukambensis*, and our results constitute the first report on the cytotoxicity, pro-apoptotic, and cell cycle arrest effects of *L. ukambensis* against colorectal cancer cells HCT-116 and HT-29.

We demonstrated by cytometry that the cytotoxicity of 1-butanol and ethyl acetate fractions of *L. ukambensis* act probably through cell cycle arrest and apoptosis pathways in colorectal cancer cells HCT-116 and HT-29. Nevertheless, as seen in the MTS assay, the cytotoxicity of these fractions is expressed more than the apoptotic cells revealed by Hoechst and cytometry assays, meaning that there are other mechanisms of action to be explored in our future research. Moreover, we will focus our next step on the isolation of the anticancer molecules in the 1-butanol and ethyl acetate fractions of *L. ukambensis.*

The pro-apoptotic effect of the most active fractions was confirmed by the significant change induced by these fractions on HCT-116 cell cycle, including a decrease in G1 and an increase in G2/M phases. It is well known that apoptosis and cell proliferation are linked by cell-cycle regulators, and that apoptotic stimuli affect both processes. Indeed some cell-cycle genes including p53, RB, and E2F are implicated in the balance between apoptosis and proliferation. This balance is strictly necessary in order to sustain tissue homeostasis. Moreover, mitosis and apoptosis show very similar morphological features that promotes to a link between apoptosis and cell cycle, even if their DNA structures display some critical differences [26]. The fact that our fractions induce significant modification of HCT-116 cell cycle opens a great perspective to elucidate the impact of the cytotoxic molecules present in these fractions on apoptosisregulating proteins, as well as cell-cycle genes.

## Conclusion

The outcome of this study suggests the interest of *L. ukambensis* for colorectal cancer management. Indeed the 1-butanol and the ethyl acetate fraction of the crude extract of this plant exhibited significant cytotoxicity against HCT-116 and HT-29 colorectal cancer cells. In addition, these fractions induced significant apoptotic and cell cycle arrest effects on HCT-116 cells. These results constitute a scientific basis for the traditional uses of *L. ukambensis* and give a great perspective for the isolation and biological investigation of natural anticancer molecules from these plant fractions.

## Figures and Tables

**Table 1:** IC_50_ values of crude extract and fractions of *L. ukambensis* on HCT-116 and HT-29 at 48H

Samples	IC_50_ (μg/mL)	
	HCT-116	HT-29
Crude extract	23.05 ± 1.56	106.81 ± 4.32
Methylene chloride fraction	5.11 ± 0.52	14.94 ± 2.98
Ethyl acetate fraction	2.41 ± 0.1	5.18 ± 0.31
1-Butanol fraction	4.32 ± 0.57	11.64 ± 0.58
